# Lobar or sublobar resection for early-stage second primary lung cancer ≤ 3 cm in size: a SEER population-based study

**DOI:** 10.1007/s00432-023-05396-5

**Published:** 2023-09-19

**Authors:** Ke Zhao, Chunqiu Xia, Minghan Qiu, Zhen Yang, Tingkai Cui, Teng Song, Shuping Li, Hanwei Mei, Yang Zheng, Huaqing Wang

**Affiliations:** 1 Department of Oncology, Tianjin Union Medical Center, Nankai University, Tianjin, 300121 China; 2 The Institute of Translational Medicine, Tianjin Union Medical Center, Nankai University, Tianjin, 300121 China; 3 Department of Lung Cancer Surgery, Tianjin Medical University General Hospital, Tianjin, 300052 China; 4 Department of Maternal, Child and Adolescence Health, School of Public Health, Tianjin Medical University, Tianjin, 300070 China

**Keywords:** Multiple primary lung cancer, Second primary lung cancer, Lobar resection, Sublobar resection, Propensity score matching

## Abstract

**Purpose:**

Surgical strategy for second primary lung cancer (SPLC) may be more conservative due to influence of first primary lung cancer (FPLC). The optimal surgical method for SPLC warrants discussion. We aimed to explore a more suitable surgical approach for early-stage (T1-T2N0, ≤ 3 cm) SPLC and provide insights for clinical practice.

**Methods:**

A retrospective study was conducted using data from the Surveillance, Epidemiology and End Results database between 2004 and 2018, and data of patients with early-stage SPLC who underwent secondary surgery were collected. Propensity score matching (PSM) reduced potential bias between lobar and sublobar resection groups. The effect of lobar and sublobar resection on overall survival (OS) was assessed in all patients and subgroups.

**Results:**

A total of 714 patients who met the study entry criteria were enrolled, including 476 patients in the sublobar resection group (66.67%) and 238 patients in the lobar resection group (33.33%). There was no difference in OS between the lobar and sublobar resection groups before and after PSM (P = 0.289) and (P = 0.608), respectively. Subgroup analyses showed that lobar resection achieved a significantly better OS than sublobar resection only in patients with an SPLC tumor size of 2–3 cm (P < 0.05).

**Conclusion:**

The OS of sublobar resection was not significantly different from that of lobar resection for early-stage SPLC. For SPLC with a 2–3 cm tumor size, lobar resection is more advantageous than sublobar resection.

**Supplementary Information:**

The online version contains supplementary material available at 10.1007/s00432-023-05396-5.

## Introduction

Multiple primary lung cancer (MPLC) is a common and special type of lung cancer that is defined as the simultaneous or sequential occurrence of two or more primary malignant tumors in the same individual (Martini and Melamed 1975). In the era of precision medicine, the survival of patients with lung cancer continues to improve with the advent of new technologies and treatment approaches. A considerable proportion of patients are diagnosed with MPLC (Siegel et al. 2021; Chiang et al. 2022). According to statistics, 0.2–20% of patients with lung cancer are diagnosed with MPLC, in which particularly common is multiple primary lung adenocarcinoma (Murphy et al. 2014; Chen et al. 2019; Rea et al. 2001; Trousse et al. 2007).

Given that most patients were diagnosed with MPLC at an early stage, surgical intervention was judged as the most predominant treatment (Waller 2018). For primary lung cancer, lobectomy with systematic lymph node dissection has been a standard surgical procedure for early-stage non-small cell lung cancer since the Lung Cancer Study Group discovery was unveiled in 1995 (Ginsberg et al. 1995). However, recent studies have reported that both lobar resection and sublobar resection showed similar efficacy in specific lung cancer subgroups. As indicated in the CALGB 140503 study (Altorki et al. 2023), sublobar resection may sufficient for ≤ 2 cm early-stage non-small cell lung cancer. Unlike primary lung cancer, underlying lung parenchyma injury and decreased pulmonary reserve after the first surgery may result in poor tolerance to secondary anatomical lobectomy in patients with MPLC, thus making the selection of surgical methods for MPLC patients relatively complex and the optimal surgical strategy remains controversial.

Second primary lung cancer (SPLC) is more common in patients with MPLC. Moreover, because of the irreversible damage to pulmonary function by each surgical intervention, patients with SPLC were more likely to undergo surgical resection than those with other types of MPLC. This study mainly aims to explore the prognostic value of lobar and sublobar resection for patients with early-stage SPLC.

## Material and methods

### Study population

**Fig. 1:**
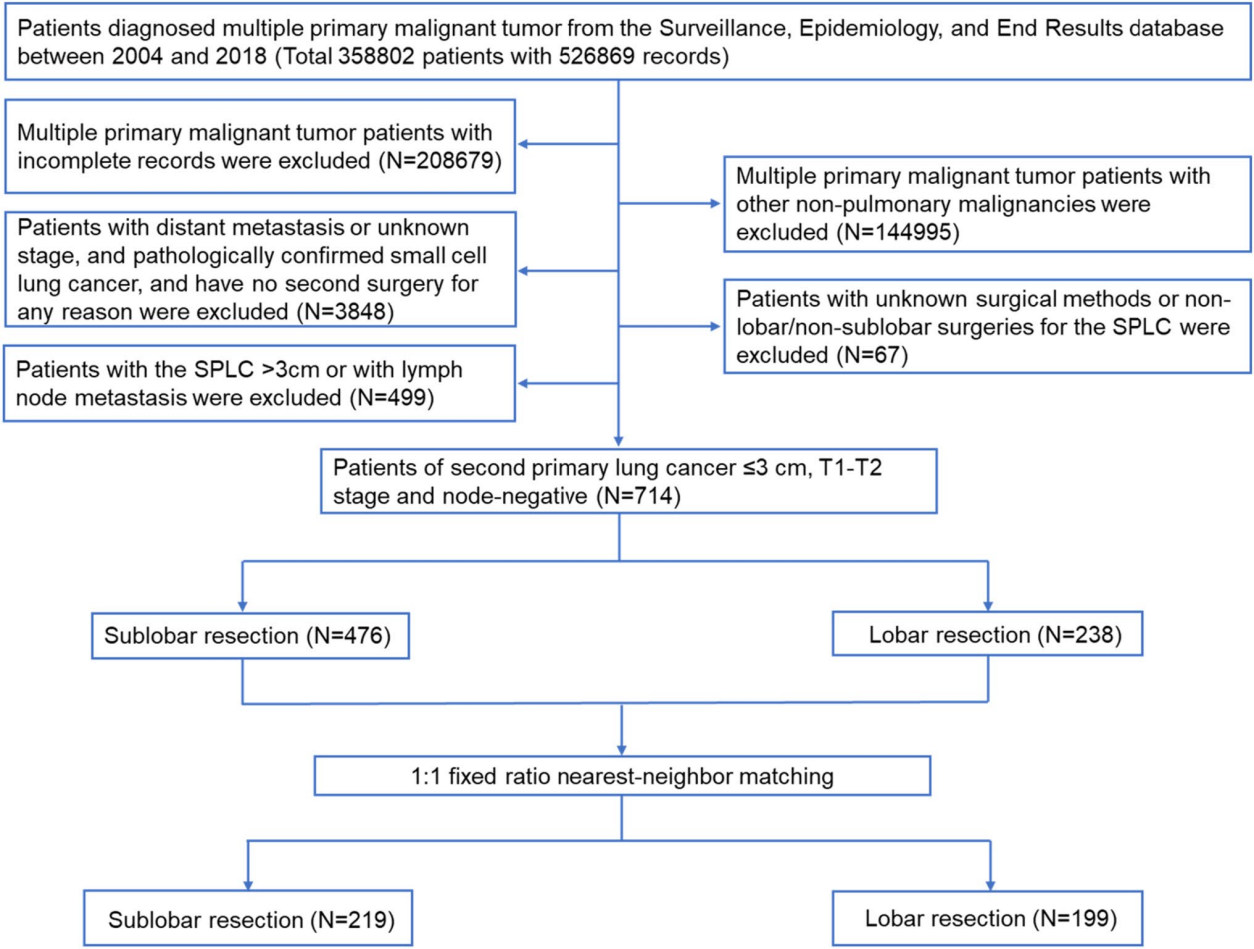
The flowchart of the screening process

We conducted a retrospective cohort study based on data from the Surveillance, Epidemiology, and End Results (SEER) database (2004–2018), and original information on patients diagnosed with multiple primary malignant tumors was collected. First, patients who were diagnosed with multiple primary tumors with incomplete records were excluded. Next, patients with multiple primary tumors other than lung cancer were excluded, meanwhile those with distant metastasis or unknown stage, and pathologically confirmed small cell lung cancer were excluded. Patients who did not undergo a second surgery for any reason were also excluded. Eventually, patients with primary lesion size of SPLC ≤ 3 cm, T1–T2 stage (pleural invasion and ≤ 3 cm), node-negative SPLC, and those who underwent lobar or sublobar resection were selected as the target population. Since the data in our study were obtained from the publicly available SEER database, ethical approval by the medical ethics committee was not required. A flowchart of the screening process is shown in Fig. 1.

### Study methods

Propensity score-matching (PSM) analysis was conducted to minimize the bias of this retrospective observational study. Based on our study aims, age at the time of SPLC diagnosis, sex, basic characteristics of the FPLC (primary site, histological grade, pathological type, tumor size, T stage, N stage, and surgical modality), baseline characteristics of SPLC (primary site, histological grade, pathological type, tumor size, T stage, and surgical modality), and whether the first and second surgeries were on the same side as possible confounders were included in the model. The two groups of patients who underwent lobar and sublobar resection were matched using nearest-neighbor matching without replacement at a fixed ratio of 1:1. The caliper was set to 0.05. The second surgery strategy of lobar and sublobar resection on overall survival (OS) of SPLC patients was compared before and after PSM. Subgroup analysis was performed according to different baseline characteristics. Multivariate analysis was performed to identify relevant prognostic factors using the Cox proportional hazards model with a P value of < 0.05 in the univariate analysis.

### Definitions

Sublobar resection was defined as wedge resection, segmental resection, other unspecified excision or resection of less than one lobe. OS was calculated from the diagnosis date of SPLC to either the date of death or the date of last follow-up. Tumor staging was converted from the previous staging in the database to the eighth edition of the Tumor Node Metastasis (TNM) staging system for lung cancer. Recurrence or second primary lung cancer was estimated by TNM stages.

### Statistical analysis

Comparisons were performed using the Chi-squared test or Fisher’s exact test for categorical variables. Survival analyses were performed using the Kaplan–Meier method and survival rates between groups were compared using log-rank test. Univariate and multivariate COX proportional hazard models were used to determine the prognostic factors associated with OS. Variables with P values < 0.05 in the univariate COX analysis were introduced into the multivariate Cox regression model. Statistical significance was set at P < 0.05. Data were analyzed using SPSS version 27.0. PSM was conducted in STATA version 17.0 using psmatch2. Survival curves and forest graph were drawn using RStudio version 4.2.1.

## Results

### Clinical characteristics of patients and tumors

The baseline clinical characteristics of the patients and tumors in the two groups are shown in Table 1. A total of 714 patients who met the inclusion and exclusion criteria were enrolled in our study, including 476 (66.67%) patients who underwent sublobar resection and 238 (33.33%) who underwent lobar resection. The baseline characteristics differed between the two groups before PSM, mainly in the surgical modality of FPLC and primary site, histological grade, T stage of SPLC, and whether the primary site of the SPLC was ipsilateral to the previous surgical site. However, the baseline difference was balanced between the two groups after PSM.

**Table 1:** Baseline clinical characteristics of patients in the lobar and sublobar resection groups before and after PSM

Characteristics	Before PSM			After PSM		
	Sub-L (n = 476)	Lob (n = 238)	P	Sub-L (n = 219)	Lob (n = 199)	P
Age (years), n (%)						
< 65	124 (26.1)	72 (30.3)	0.236	67 (30.6)	56 (28.1)	0.583
≥ 65	352 (73.9)	166 (69.7)		152 (69.4)	143 (71.9)	
Sex, n (%)						
Female	304 (63.9)	144 (60.5)	0.381	136 (62.1)	120 (60.3)	0.706
Male	172 (36.1)	94 (39.5)		83 (37.9)	79 (39.7)	
Side of first and second surgery, n (%)						
Ipsilateral	177 (37.2)	141 (59.2)	< 0.001	107 (48.9)	102 (51.3)	0.624
Contralateral	299 (62.8)	97 (40.8)		112 (51.1)	97 (48.7)	
Surgical modality^a^, n (%)						
Sublobar resection	162 (34.0)	31 (13.0)	< 0.001^*^	51 (23.3)	31 (15.6)	0.050^*^
Lobar resection	305 (64.1)	204 (85.7)		161 (73.5)	166 (83.4)	
Pneumonectomy	7 (1.5)	3 (1.3)		5 (2.3)	2 (1.0)	
Other modalities	2 (0.4)	0 (0.0)		2 (0.9)	0 (0.0)	
Primary site^a^, n (%)						
Upper lobe	311 (65.3)	153 (64.3)	0.781	147 (67.1)	121 (60.8)	0.179
Non-upper lobe	165 (34.7)	85 (35.7)		72 (32.9)	78 (39.2)	
Primary site^b^, n (%)						
Upper lobe	231 (48.5)	141 (59.2)	0.007	126 (57.5)	111 (55.8)	0.718
Non-upper lobe	245 (51.5)	97 (40.8)		93 (42.5)	88 (44.2)	
Histological grade^a^, n (%)						
G1	88 (18.5)	45 (18.9)	0.308^*^	43 (19.6)	41 (20.6)	0.887^*^
G2	198 (41.6)	99 (41.6)		88 (40.2)	85 (42.7)	
G3	140 (29.4)	69 (29.0)		68 (31.1)	56 (28.1)	
G4	12 (2.5)	1 (0.4)		3 (1.4)	1 (0.5)	
Unknown	38 (8.0)	24 (10.1)		17 (7.8)	16 (8.0)	
Histological grade^b^, n (%)						
G1	139 (29.2)	68 (28.6)	0.028^*^	60 (27.4)	60 (30.2)	0.714^*^
G2	191 (40.1)	93 (39.1)		80 (36.5)	79 (39.7)	
G3	91 (19.1)	33 (13.9)		44 (20.1)	30 (15.1)	
G4	6 (1.3)	1 (0.4)		1 (0.5)	1 (0.5)	
Unknown	49 (10.3)	43 (18.1)		34 (15.5)	29 (14.6)	
Pathological type^a^, n (%)						
Squamous cell carcinoma	102 (21.4)	56 (23.5)	0.608	55 (25.1)	42 (21.1)	0.187
Adenocarcinoma	253 (53.2)	129 (54.2)		107 (48.9)	115 (57.8)	
Other types	121 (25.4)	53 (22.3)		57 (26.0)	42 (21.1)	
Pathological type^b^, n (%)						
Squamous cell carcinoma	90 (18.9)	43 (18.1)	0.633	39 (17.8)	39 (19.6)	0.895
Adenocarcinoma	264 (55.5)	126 (52.9)		120 (54.8)	107 (53.8)	
Other types	122 (25.6)	69 (29.0)		60 (27.4)	53 (26.6)	
T stage^a^, n (%)						
T1	250 (52.5)	135 (56.7)	0.324^*^	110 (50.2)	110 (55.3)	0.089^*^
T2	133 (27.9)	71 (29.8)		57 (26.0)	63 (31.7)	
T3	47 (9.9)	19 (8.0)		29 (13.2)	15 (7.5)	
T4	36 (7.6)	11 (4.6)		18 (8.2)	9 (4.5)	
Tx	10 (2.1)	2 (0.8)		5 (2.3)	2 (1.0)	
T stage^b^, n (%)						
T1	409 (85.9)	220 (92.4)	0.011	198 (90.4)	181 (91.0)	0.849
T2	67 (14.1)	18 (7.6)		21 (9.6)	18 (9.0)	
N stage^a^, n (%)						
N0	390 (81.9)	210 (88.2)	0.170^*^	181 (82.6)	179 (89.9)	0.055^*^
N1	47 (9.9)	14 (5.9)		23 (10.5)	8 (4.0)	
N2	34 (7.1)	13 (5.5)		13 (5.9)	11 (5.5)	
Nx	5 (1.1)	1 (0.4)		2 (0.9)	1 (0.5)	
Tumor size^a^, n (%)						
≤ 2 cm	205 (43.1)	105 (44.1)	0.666^*^	91 (41.6)	89 (44.7)	0.627^*^
2–3 cm	127 (26.7)	67 (28.2)		53 (24.2)	52 (26.1)	
> 3 cm	137 (28.8)	65 (27.3)		72 (32.9)	57 (28.6)	
Unknown	7 (1.5)	1 (0.4)		3 (1.4)	1 (0.5)	
Tumor size^b^, n (%)						
≤ 2 cm	405 (85.1)	190 (79.8)	0.076	186 (84.9)	161 (80.9)	0.274
2–3 cm	71 (14.9)	48 (20.2)		33 (15.1)	38 (19.1)	

*Sub-L* Sublobar resection, *Lob* Lobar resection

^a^First primary lung cancer

^b^Second primary lung cancer

*Fisher’s exact test

### Comparison of survival in all patients

**Fig. 2:**
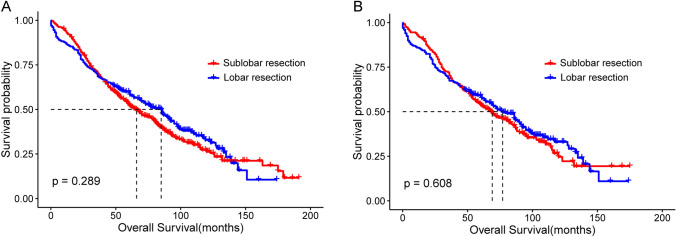
Kaplan–Meier survival curves of all patients between the groups divided by the surgical modality of SPLC. **A** Comparison between sublobar and lobar resection in all patients before PSM. **B** Comparison between sublobar and lobar resection in all patients after PSM

Differences in the OS of all patients were analyzed using the log-rank test. Results before PSM showed that OS in the lobar resection group was better than that of the sublobar resection group; however, there was no significant difference between the two groups (median OS: 85.0 m vs. 66.0 m; P = 0.289, Fig. 2A). After PSM, a comparison of OS still showed no significant difference between the patients in lobar and sublobar resection groups (median OS: 77.0 m vs. 69.0 m; P = 0.608, Fig. 2B).

### Comparison of patients’ survival in different subgroups

**Fig. 3:**
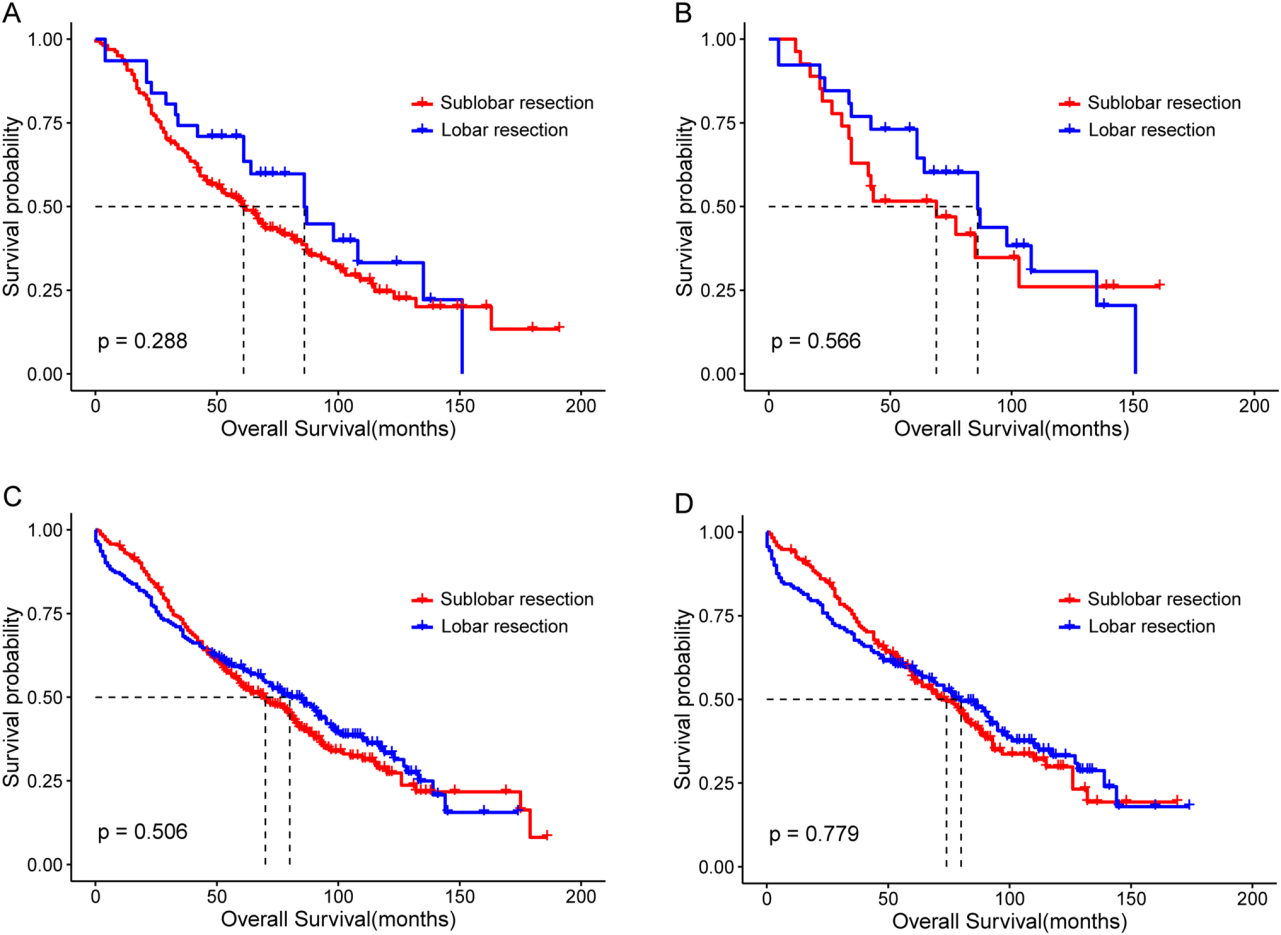
Kaplan–Meier survival curves of the sublobar and lobar resection groups of patients with SPLC stratified by the surgical modality of FPLC. **A** Patients with FPLC underwent sublobar resection before PSM. **B** Patients with FPLC underwent sublobar resection after PSM. **C** Patients with FPLC underwent lobar resection before PSM. **D** Patients with FPLC underwent lobar resection after PSM

To explore whether the overall data masked the divergence of OS in a specific subgroup, we divided the patients into four subgroups. Patients were grouped according to the surgical modality of FPLC. In the sublobar resection subgroup (n = 193), though patients with SPLC who underwent lobar resection had a better OS than those who underwent sublobar resection, there were no significant differences before PSM (median OS: 86.0 m vs. 61.0 m; P = 0.288, Fig. 3A) and after PSM (median OS: 86.0 m vs. 69.0 m; P = 0.566, Fig. 3B). In the lobar resection subgroup (n = 509), OS was similar between the lobar and sublobar resection groups before PSM (median OS: 80.0 m vs. 70.0 m; P = 0.506, Fig. 3C) and after PSM (median OS: 80.0 m vs. 74.0 m; P = 0.779, Fig. 3D).

**Fig. 4:**
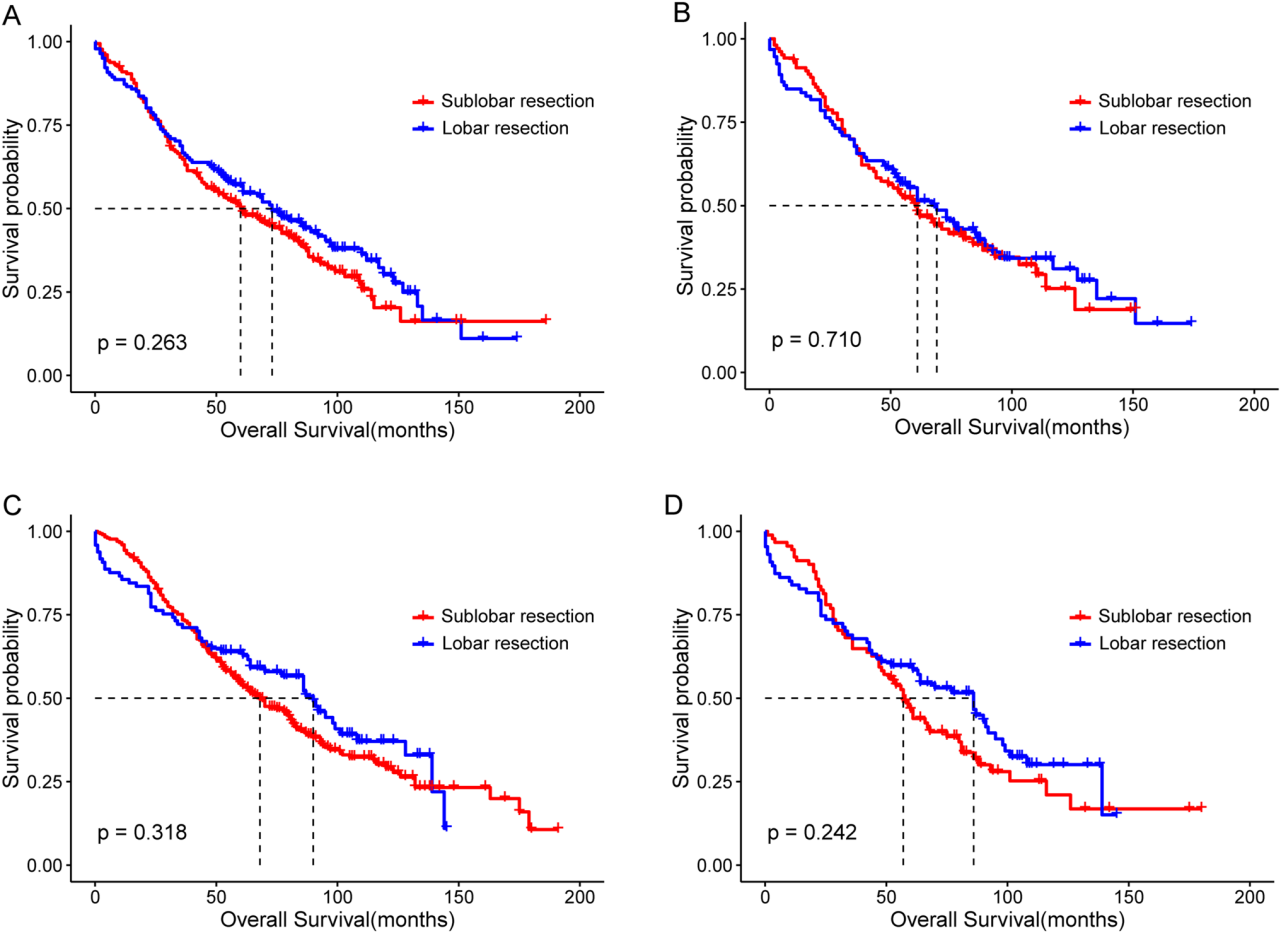
Kaplan–Meier survival curves of the sublobar and lobar resection groups of patients with SPLC stratified by the side of the first and second surgery. **A** Ipsilateral subgroup before PSM. **B** Ipsilateral subgroup after PSM. **C** Contralateral subgroup before PSM. **D** Contralateral subgroup after PSM

Based on the use of the same or different sides between the first and second surgeries, we divided the patients into the ipsilateral (n = 318) and contralateral (n = 396) subgroups. Analysis by the log-rank test showed that patients with SPLC who underwent lobar resection had a slightly better OS than those who underwent sublobar resection; however, there were no statistically significant differences between the ipsilateral or contralateral subgroups. The differences in OS in the ipsilateral subgroup before and after matching are shown in Fig. 4A (median OS: 73.0 m vs. 60.0 m; P = 0.263) and 4B (median OS: 69.0 m vs. 61.0 m; P = 0.710), respectively. Also, the similar result in the contralateral subgroup before and after matching are shown in Figs. 4C (median OS: 90.0 m vs. 68.0 m; P = 0.318) and 4D (median OS: 86.0 m vs. 57.0 m; P = 0.242), respectively.

**Fig. 5:**
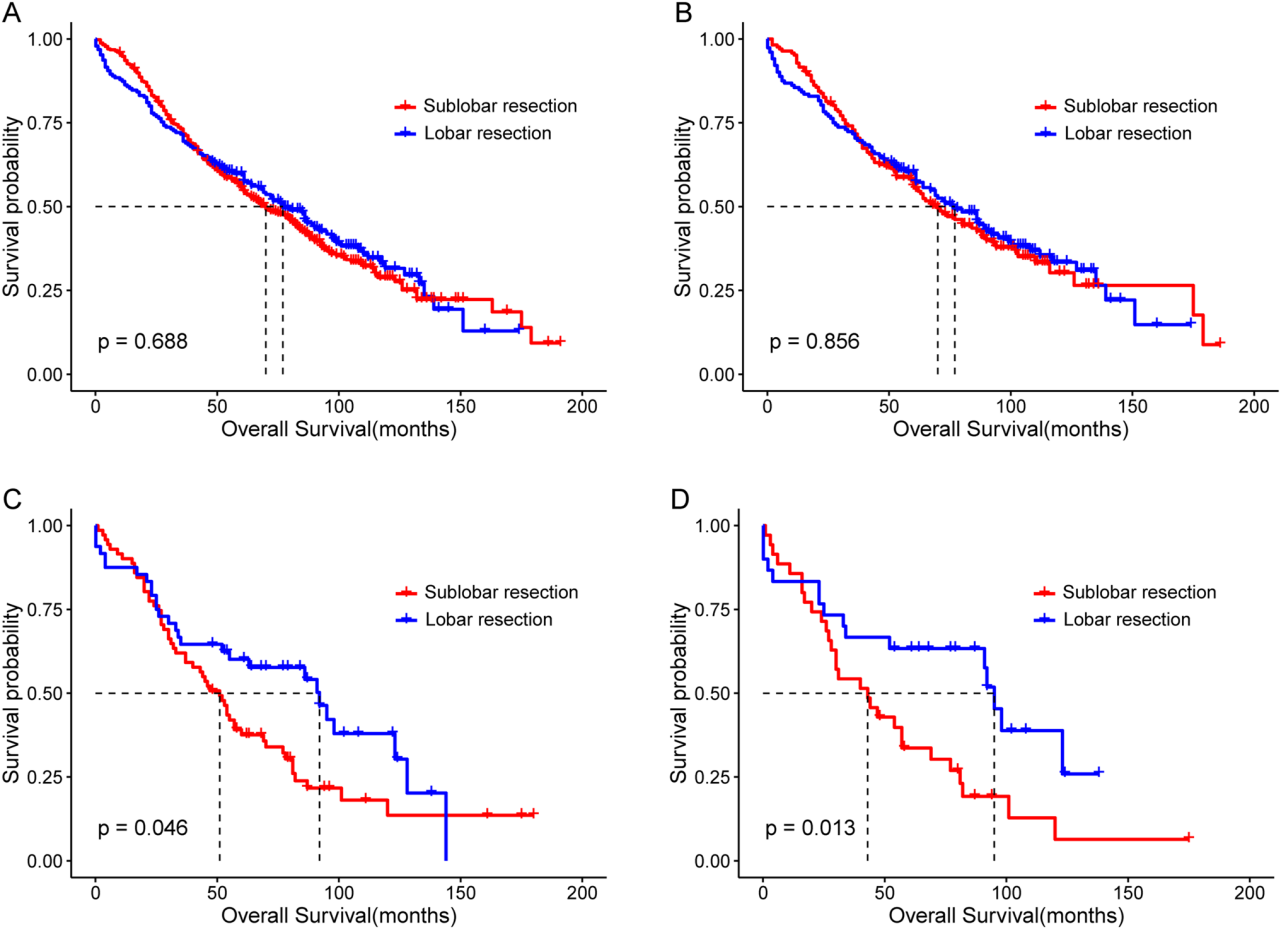
Kaplan–Meier survival curves of the sublobar and lobar resection groups of patients with SPLC stratified by the primary tumor size of SPLC. **A** The ≤ 2 cm subgroup before PSM. **B** The ≤ 2 cm subgroup after PSM. **C** The 2–3 cm subgroup before PSM. **D** The 2–3 cm subgroup after PSM

Based on the primary tumor size in SPLC, patients were classified into the ≤ 2 cm subgroup (n = 595) and the 2–3 cm subgroup (n = 119). In the ≤ 2 cm subgroup, the prognosis was similar in the lobar and sublobar resection groups, with no significant difference in OS before PSM (median OS: 77.0 m vs. 70.0 m; P = 0.688, Fig. 5A) and after PSM (median OS: 77.0 m vs. 70.0 m; P = 0.856, Fig. 5B). However, in the 2–3 cm subgroup, lobar resection was significantly better than sublobar resection in OS before PSM (median OS: 92.0 m vs. 51.0 m; P = 0.046, Fig. 5C), and this trend was more pronounced after PSM (median OS: 95.0 m vs. 43.0 m; P = 0.013, Fig. 5D).

**Fig. 6:**
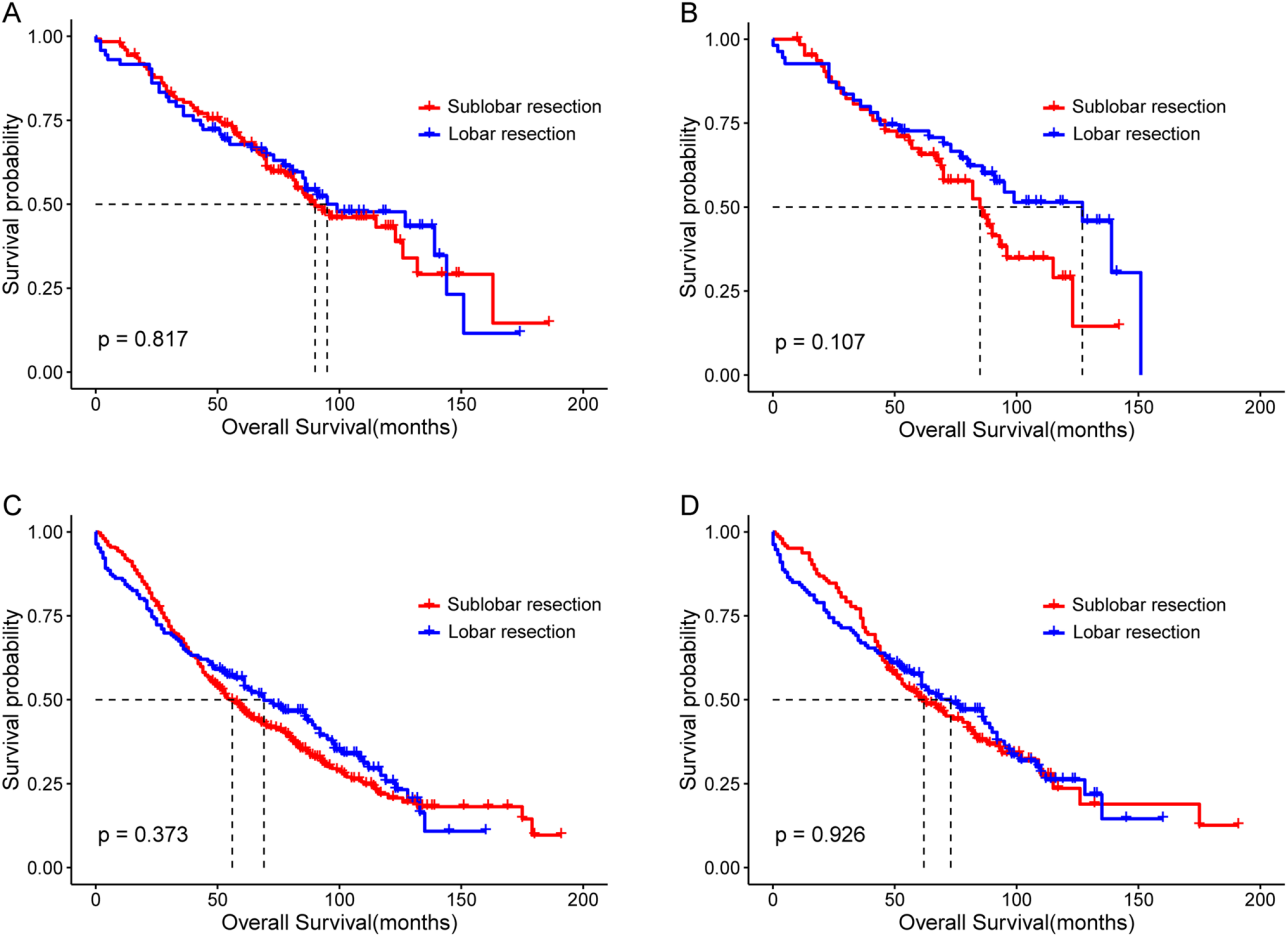
Kaplan–Meier survival curves of the sublobar and lobar resection groups of patients with SPLC stratified by the age at SPLC diagnosis. **A** The < 65 years subgroup before PSM. **B** The < 65 years subgroup after PSM. **C** The ≥ 65 years subgroup before PSM. **D** The ≥ 65 years subgroup after PSM

Furthermore, we divided the patients into either the < 65 years or ≥ 65 years subgroups based on age at SPLC diagnosis. The log-rank test revealed that the differences in OS between the lobar and sublobar resection groups were not significant in any age subgroup. OS in the < 65 years subgroup before and after matching are shown in Figs. 6A (median OS: 95.0 m vs. 90.0 m; P = 0.817) and 6B (median OS: 127.0 m vs. 85.0 m; P = 0.107), respectively. Figures 6C (median OS: 69.0 m vs. 55.0 m; P = 0.373) and 6D (median OS: 73.0 m vs. 62.0 m; P = 0.926), respectively, present the OS differences in the ≥ 65 years subgroup before and after matching.

**Fig. 7:**
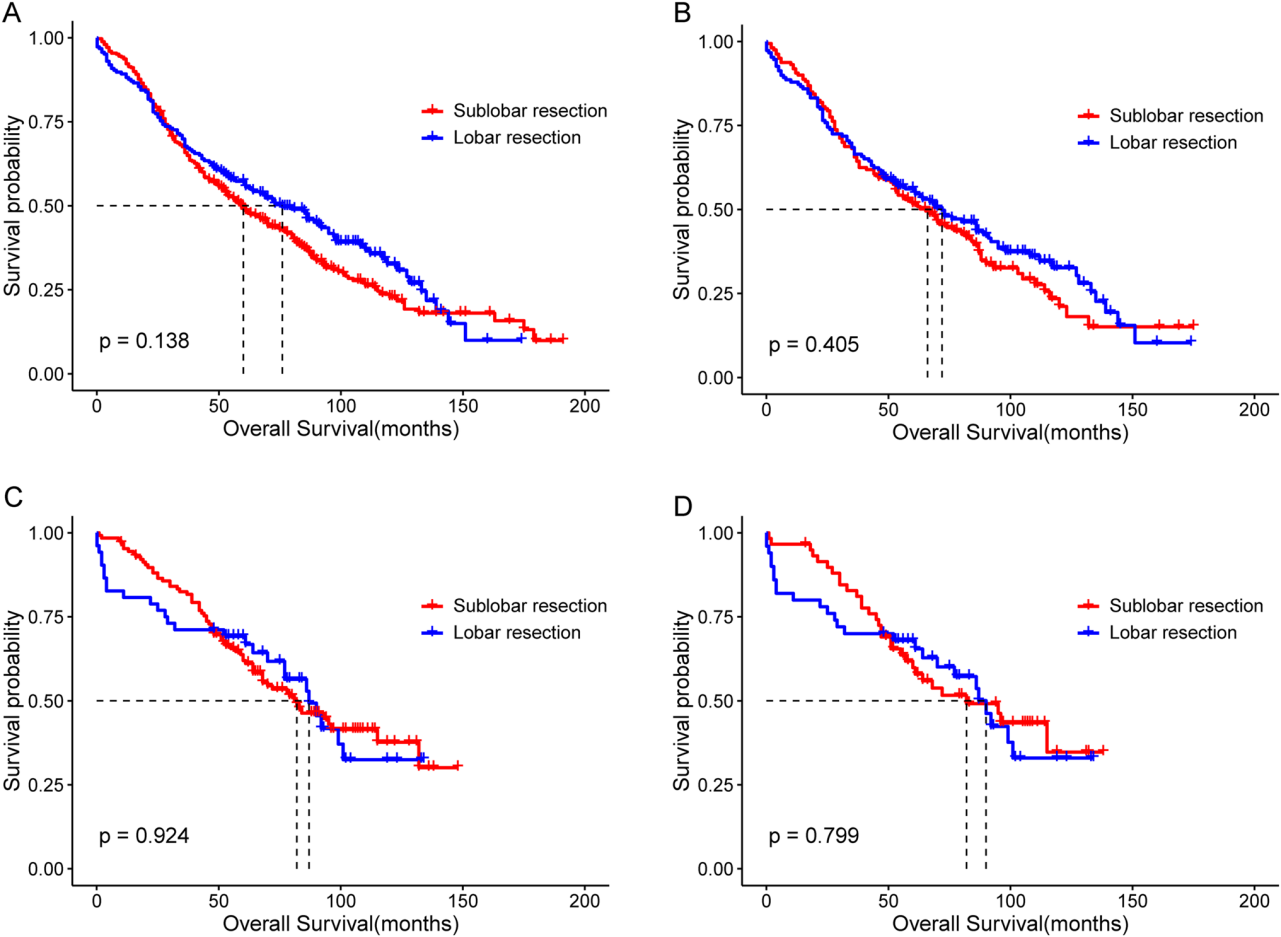
Kaplan–Meier survival curves of the sublobar and lobar resection groups of patients with SPLC stratified by the time interval between FPLC and SPLC. **A** The sMPLC subgroup before PSM. **B** The sMPLC subgroup after PSM. **C** The mMPLC subgroup before PSM. **D** The mMPLC subgroup after PSM

Patients were categorized into synchronous multiple primary lung cancer (sMPLC, n = 535) or metachronous multiple primary lung cancer (mMPLC, n = 179) subgroups based on the diagnosis time interval between FPLC and SPLC (≤ 2 years vs. > 2 years) (Martini and Melamed 1975b). Analysis by the log-rank test showed that patients with SPLC who underwent lobar resection had a slightly better OS than those who underwent sublobar resection; however, there were no statistically significant differences between the sMPLC or mMPLC subgroups. The differences in OS in the sMPLC subgroup before and after matching are shown in Fig. 7A (median OS: 60.0 m vs. 76.0 m; P = 0.138) and 7B (median OS: 66.0 m vs. 72.0 m; P = 0.405), respectively. Also, the similar result in the mMPLC subgroup before and after matching are shown in Figs. 7C (median OS: 82.0 m vs. 87.0 m; P = 0.924) and 7D (median OS: 82.0 m vs. 90.0 m; P = 0.799), respectively.

### Univariate and multivariate Cox regression analyses for selecting prognostic factors

**Fig. 8:**
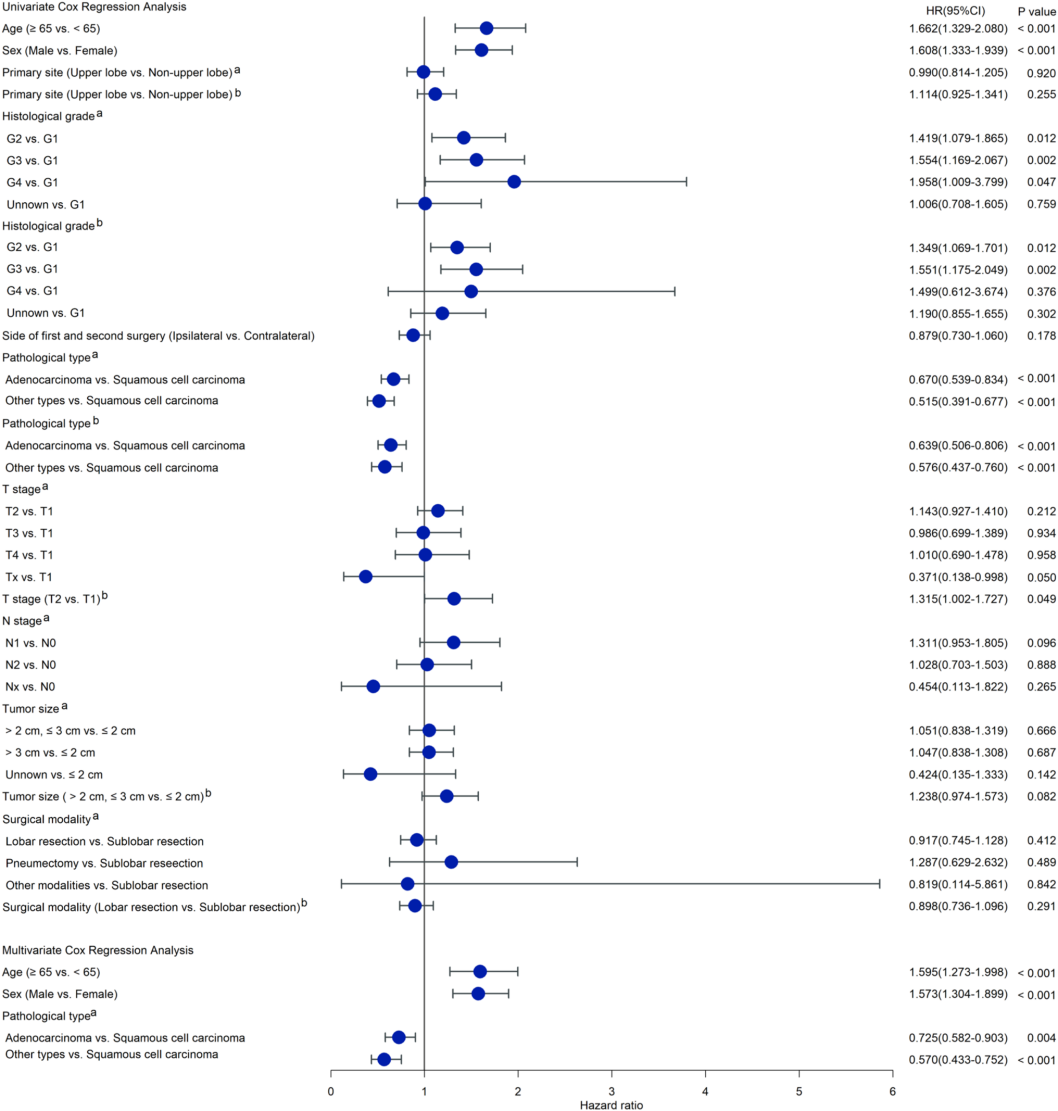
Univariate and multivariate COX regression analyses of prognostic factors for OS

Univariate Cox analysis revealed that age at SPLC diagnosis, sex, histological grade, and pathological type of FPLC, as well as histological grade, pathological type, and T stage of SPLC, were prognostic factors influencing survival outcome. However, the primary lesion location of FPLC and SPLC, tumor size of FPLC and SPLC, N stage of FPLC, whether the first and second surgery was performed on the same side, and surgical modality of FPLC and SPLC were not significantly associated with survival. Multivariate Cox regression analysis was performed by incorporating significant factors (P < 0.05) in the univariate Cox model. We demonstrated that age ≥ 65 years, male sex, and pathologically confirmed squamous cell carcinoma of the FPLC were associated with worse survival (Fig. 8).

## Discussion

We focused on the selection of a secondary surgical modality in patients with SPLC and mainly explored the effect of surgery strategy, lobar resection, or sublobar resection on the OS of patients with early-stage SPLC in this study. In the overall study population, lobar resection did not prove to be significantly better than sublobar resection before and after PSM. To explore whether the overall data masked the divergence of OS in a certain subgroup, patients were grouped into subclusters by age, surgical modality of FPLC, tumor size of SPLC, and whether the first and second surgeries were on the same side. Eventually, we observed that OS was higher among patients who underwent lobar resection than those who underwent sublobar resection only in the SPLC subgroup with a tumor size of 2–3 cm, whereas no significant differences were observed in survival among the remaining subgroups.

Previously, the surgical modality of MPLC was mostly lobar resection or pneumonectomy. However, sublobar resection has progressively become more popular in recent years (Zhao et al. 2020). Previous studies have reported that lobar resection is superior to sublobar resection and should be regarded as the preferred treatment for patients with SPLC (Zuin et al. 2013). It has also been argued that the survival rate of SPLC patients who underwent sublobar resection was no less than that of those who underwent lobar resection (Bae et al. 2011; Lee et al. 2019; Wu et al. 2020; Zhao et al. 2017), especially patients with poor pulmonary function, who are considered safer to undergo sublobar resection (Lee et al. 2019; Yang et al. 2016). A pooled meta-analysis of 10 independent studies revealed that the OS of patients with MPLC who underwent sublobar resection was comparable to that of patients who achieved complete and standard resection (lobar resection or pneumonectomy). Even the application of a relatively conservative surgical strategy has no negative impact on the prognosis of lung cancer patients with poor pulmonary function (Chen et al. 2019). The conclusions of the studies mentioned above are not uniform and may be attributed to differences in the diagnostic criteria and study population. Furthermore, the majority of studies only focused on exploring the tumor characteristics of SPLC and ignored the potential impact of the characteristics of FPLC on OS, making the study results relatively limited. Contrary to previous studies, we used PSM to balance the differences in baseline characteristics of FPLC and SPLC to minimize the impact of selection bias on the results as much as possible in this study. Ultimately, we found that limited sublobar resection was acceptable for SPLC with a diameter of ≤ 3 cm. The results of our study may broaden the range of potential candidates for surgery, enabling more patients to be eligible for better-tolerated surgical treatment and provide clinicians with a greater choice of surgical methods.

The clinical characteristics and comorbidities of patients are extremely important for the formulation of a surgical strategy; therefore, we propose that the surgical methods for patients with SPLC should be individualized. In the subgroup analyses of this study, for tumors with a diameter ≤ 2 cm, regardless of whether the FPLC patients were treated with sublobar or lobar resection, whether the first surgery was ipsilateral to the second surgery or contralateral, the age distribution of patients, or synchronicity of the two cancers, sublobar resection was of high value and showed equivalence with lobar resection. According to Schwartz et al. (Schwartz et al. 2016), the quality of life of patients who underwent wedge resection surgery was superior to lobar resection. This was mainly due to the lower total number of lung segments resected by wedge resection surgery, which probably promoted the recovery of residual pulmonary function. Thus, sublobar resection may be a better alternative for patients with combined chronic obstructive pulmonary disease, asthma, or other comorbidities. Conversely, lobar resection significantly improved survival in patients with SPLC and a tumor size of 2–3 cm.

From multivariate Cox regression analysis, age ≥ 65 years, male sex, and squamous cell carcinoma of FPLC were considered independent predictors of poor OS in patients with SPLC. A potential explanation for the lack of significance of FPLC staging in the multivariate analysis is that most patients receiving surgery for SPLC tend to have earlier stage first lung cancers that were well controlled. In this study cohort, first primary lung cancers were predominantly stage I (68.3%), which may have minimized any potential survival impact on the analysis. Previous studies have also reported that older age, male sex, and pathologically diagnosed squamous cell carcinoma showed an even poorer oncologic outcome, whereas the tumor size of SPLC had no significant effect on prognosis and survival (Lee et al. 2019). Therefore, in patients with poor prognoses, sublobar resection with less trauma is likely to bring an ever-better quality of life. Furthermore, it is noteworthy that the surgical modality of FPLC did not significantly affect the choice of surgical approach for patients with SPLC in our study, which differs from our preconception. This may be due to a lack of data such as pulmonary function, and we failed to analyze the effect of the surgical modality of FPLC on patients. Currently, the surgical modality of FPLC as a stratified factor to explore the difference in OS of patients with SPLC who underwent lobar or sublobar resection has not been reported in the literature. Also, the effect of the superposition of both surgeries on patient prognosis awaits further study.

Our study was based on the SEER database and had the advantages of a large sample size. We also ensured the study was objective and detailed, using real-world clinical data. However, there were some limitations in our study. The first limitation is the study’s retrospective design. Although some confounding factors were matched by PSM to reduce their influence on the conclusion, other potential confounders may have affected the analysis results. Second, the lack of data on cardiopulmonary performance, comorbidities, adjuvant chemotherapy, or targeted treatment makes it difficult to analyze the impact of these clinical high-risk factors on the surgical strategy. In addition, due to limited data collection, we only analyzed the prognostic value of different secondary surgical approaches for SPLC, and the choice of surgical modality in patients who underwent surgery three or more times was not discussed.

In conclusion, sublobar resection was comparable to lobar resection in patients with SPLC who underwent secondary surgery. For patients with SPLC and a tumor size of 2–3 cm, lobar resection may result in better survival. These findings require further validation in future prospective trials.

## Supplementary Information

Below is the link to the electronic supplementary material.

Supplementary file1 (XLSX 29785 KB)

Supplementary file2 (XLSX 29774 KB)

Supplementary file3 (XLSX 29798 KB)

Supplementary file4 (XLSX 29788 KB)

Supplementary file5 (XLSX 29752 KB)

Supplementary file6 (XLSX 7929 KB)

## Data Availability

The authors confirm that the data supporting the findings of this study are available within the article in supplementary information.
